# Opportunities and Challenges for Incorporating Artificial Intelligence and Natural Language Processing in Neurology Education

**DOI:** 10.1212/NE9.0000000000200116

**Published:** 2024-02-27

**Authors:** Renzo Figari Jordan, Stefano Sandrone, Andrew M. Southerland

**Affiliations:** From the Department of Neurology (R.F.J., A.M.S.), University of Virginia, Charlottesville; and Division of Neuroscience (S.S.), Imperial College London, United Kingdom.

Since the launch of OpenAI's ChatGPT, the application of artificial intelligence (AI) in medical education has ignited a polarized discussion. Two main advancements will change the way we study and practice medicine, large language models (LLMs), such as generated pretrained transformer 4 (GPT-4), and natural language processing (NLP). While LLM performs language-related tasks, NLP enables machines to understand and generate human language.

LLM and NLP allow neurology learners to access and synthesize information, albeit with some considerations. Medical learners struggle with an exponentially expanding medical knowledge, doubling every 73 days.^1^ Internships are “training in the art of culling data.”^2^ Neurology's complexity, compounded by “neurophobia,” underlines the need for efficacious knowledge acquisition and clinical application.^3^

However, concerns arise regarding the trustworthiness of NLP-generated information. ChatGPT, for example, uses multiple sources of data sets obtained from the internet, such as Wikipedia and Reddit, wth early versions limited to knowledge up to 2021. NLP is programmed not to lie, yet these programs are known to “confabulate” and “hallucinate” to answer prompts. Learners and educators should know that NLP culls from a data set, bases recommendations from these input sources, and avoids biased decision making, but does not display inherent bias that exists in the data. AI assistance should be used carefully to protect patients' information. DocsGPT is one of the first Health Insurance Portability and Accountability Act (HIPAA)-compliant AI assistance that is freely available through Doximity.

On the positive side, imagine an NLP with access to all medical journals, summarizing the latest research on specific topics in minutes using the preferred medium, that is, written, audio, or video. Newer NLP tools, such as those created by John Snow Labs, are already capable of summarizing medical records and providing efficient data. NLP can also provide accurate data summarization of referral notes, charts, or laboratory results. It can write documentation, send result letters, and respond to insurance company requests. This enhances efficiency and allows neurologists and trainees to spend time with patients or in learning. The responses generated by ChatGPT are nearly indistinguishable from those provided by physicians.^4^ The US Food and Drug Administration has approved 14 AI technologies for clinical neurologic applications.^5^

In a survey of medical students, most are aware of the importance of this new technology. Still, because of a lack of training and exposure, most feel unqualified for a future in AI-assisted medicine.^6^ Over 70% of medical students agreed that medical education should include training in AI applications.^7^ A curriculum for undergraduate and graduate medical education has been proposed, and it should include conceptual foundations of predictive analytics, AI in neurology, and the impact of AI in health care.^8^ These tools should remain open and free to be accessible to all students and trainees, particularly underrepresented groups and those practicing in low-income countries. Rapid access to HIPAA-compliant AI-assisted diagnostic tools could bring care to remote areas lacking specialized services, such as using automated fundus photography to identify papilledema in patients with acute headaches.^9^ Family Educational Rights and Privacy Act–compliant platforms could enhance assessment and allow educators to incorporate more objective clinically based evaluations of student documentation, clinical reasoning, and other difficult-to-assess skills.

The 2019 Council Report on AI from the American Medical Association recommends involving engineers and data scientists in curriculum workgroups.^10^ Medical schools should implement AI introductory courses early in medical education, and neurology and neuroscience curricula should include modules on AI applications in neurologic practice. It will provide medical students with a foundational understanding of AI and machine learning applications in health care, which can then be integrated further within their specialty as a resident and fellow, including in neurology. This prepares the next generation of physicians to use AI tools intelligently and contribute to their development (Table).

**Table:** Key Aspects Where AI Can Revolutionize the Field, From Diagnostic Evaluation to Admission Decisions, While Also Acknowledging Potential Pitfalls That Demand Careful Consideration

Opportunities	Challenges
**Diagnostic evaluation and evidence-based medicine**	
Improved accuracy and efficiency in diagnosis	Potential risk of data “hallucinations”
Enhanced evidence-based treatment plans	Dependency on AI may lead to neglect of clinical expertise
Access to vast medical knowledge databases	
**Therapeutic decision making**	
Personalized treatment plans based on data	Lack of accountability in decision making
Real-time monitoring of patient responses	Ethical concerns around AI-driven treatment decisions
Integration of latest research findings	Resistance to AI recommendations from health care professionals
**Soft skill learning**	
Virtual simulation for interpersonal skills	Overemphasis on technical skills at the expense of soft skills
Role-playing scenarios for communication	Varied individual response to virtual scenarios
Feedback and improvement in non-technical skills	
**Clinical and learning efficiency**	
Streamlined learning processes	Potential erosion of clinical skills
Adaptive learning pathways for individual needs	Resistance to AI integration from traditional education systems
Increased efficiency in clinical workflows	
**Student assessment and evaluation**	
Objective and standardized assessment methods	Concerns about data security
Continuous monitoring of student performance	Compliance with FERPA standards
Immediate feedback for improvement	AI-based assessments may not capture holistic student abilities
**Admission and selection decisions**	
Data-driven decision making for admissions	Limited data sets for decision making
Identification of potential based on analytics	Amplification of existing biases in admission processes
Increased fairness in selection processes	Ethical dilemmas in using AI for high-stake decisions
**Ethics, equity, and inclusion**	
Identify and address biases	Risk of biased data sets affecting decisions
Promotes equity in education and opportunities	Ensuring diverse representation in AI development
Tools for ensuring inclusive learning environments	

Abbreviations: AI = artificial intelligence; FERPA = Family Educational Rights and Privacy Act.

In summary, there are 2 critical implications of AI for neurology education. First, educators should familiarize themselves with AI NLP concepts and embrace them as part of the discussion instead of banning them from the learning environment. Guidelines exist for using NLP in scholarly publications.^11^ We are now responsible for setting up strategies for our students and trainees in neurology to understand the principles of AI development and the utility and limitations of applying NLP tools such as GPT algorithms in clinical practice and teaching. This includes balancing the ethical and legal issues to consider in relying on AI-supported decision making. Second, we should research the use of NLP tools in documentation efficiency, particularly during residency, potentially avoiding the prolific copy-and-paste usage of the current electronic medical record.

Neurology educators should adapt to this technology, ensuring its safe and appropriate use by our students and trainees, and testing its efficacy to help us optimize neurologic care and education. As a community of neurology educators, we must find ways to embrace the new AI-powered technology in a safe, transparent, and creative manner, one prompt at a time.

## Appendix Authors

**Table:**

Name	Location	Contribution
Renzo Figari Jordan, MD	Department of Neurology, University of Virginia, Charlottesville	Study concept or design
Stefano Sandrone, PhD	Division of Neuroscience, Imperial College London, United Kingdom	Drafting/revision of the manuscript for content, including medical writing for content
Andrew M. Southerland, MD	Department of Neurology, University of Virginia, Charlottesville	Drafting/revision of the manuscript for content, including medical writing for content; study concept or design
